# Improving access to help with poor sleep across youth mental health services: Interim implementation and clinical outcomes

**DOI:** 10.1111/bjc.12531

**Published:** 2025-03-14

**Authors:** Rebecca Rollinson, Ben Ewing, Sarah Reeve, Adam Graham, Jonathan Lyons, Brioney Gee, Jonathon Wilson, Ioana Tofan, Kelly Semper, Tim Clarke

**Affiliations:** ^1^ Norfolk and Suffolk NHS Foundation Trust and University of East Anglia Norwich UK; ^2^ Norfolk and Suffolk NHS Foundation Trust Norwich UK; ^3^ University of East Anglia Norwich UK; ^4^ Cambridgeshire and Peterborough NHS Foundation Trust Cambridgeshire UK; ^5^ Norfolk and Waveney Integrated Care Board Norwich UK

**Keywords:** CBTi, EPIS, implementation, insomnia, mental health, sleep, youth

## Abstract

**Objectives:**

There is a high, unmet sleep need in young people with mental health difficulties. We took a whole‐system approach to improving access to sleep support across a youth mental health system (14–25 years).

**Methods:**

We used the Exploration, Preparation, Implementation and Sustainment (EPIS) framework to develop an implementation programme (The Better Sleep Programme) incorporating two levels of training: (i) therapeutic practitioners received training and supervision in CBT for insomnia (CBTi) adapted for young people with mental health difficulties, (ii) non‐therapeutic practitioners received knowledge and skills workshops. Implementation and clinical outcome measures were collected.

**Design:**

Implementation outcomes of acceptability, adoption, appropriateness, accessibility and fidelity were considered for the programme and CBTi intervention within it. Clinical outcomes for the CBTi intervention covered sleep, wellbeing and personal goals and were evaluated using a pre‐post comparison within‐subject design.

**Results:**

High levels of attendance and uptake were seen for CBTi training (210 therapeutic practitioners from 18 services) and workshops (270 attendees from 29 services). Five of the six core service areas trained were routinely offering the CBTi intervention. Significant improvements were seen across all clinical outcome measures (*n* = 83, *p* ≤ 0.001 to *p* ≤ 0.05) with moderate to large effect sizes observed across measures of sleep (*d* = 0.61–1.35), mental health (*d* = 0.57–1.26) and personal goals (*d* = 1.77).

**Conclusions:**

This centrally‐funded, system‐wide implementation programme shows significant promise as a means of improving sleep in young people with mental health difficulties. High uptake with encouraging clinical outcomes was seen across services. Further evaluation is required to establish sustainability and generalizability.

**Practitioner points**

- High levels of insomnia are being seen in young people with mental health difficulties.
- A system‐wide, centrally‐funded implementation strategy saw high engagement from youth mental health services wanting to deliver an evidence‐based sleep intervention.
- Positive clinical outcomes were seen in both sleep and wellbeing in the young people receiving a sleep intervention.

## INTRODUCTION

There is a widespread lack of availability of cognitive behavioural therapy for Insomnia (CBTi) (Baglioni et al., 2020; Koffel et al., 2018) despite its strong evidence base (Edinger et al., 2021; Van Straten et al., 2018) and status as a first‐line recommended intervention for insomnia in the United Kingdom, European Union and United States (National Institute of Health and Care Excellence, 1999, 2024; Qaseem et al., 2016; Riemann et al., 2017). There is hope that digital therapies can help improve access (Espie et al., 2019) but these are not currently routinely available in the UK National Health Service and are designed to target adult sleep difficulties rather than the specific issues faced by teenagers (Crowley et al., 2018).

This implementation gap is particularly acute in relation to treating insomnia in mental health services (Freeman et al., 2020; Harvey, 2022; Stafford et al., 2024). In part, the complexity and comorbidity of sleep difficulties can present a particular challenge (Harvey, 2022; Reeve et al., 2019) but more fundamentally, sleep is not seen as a priority treatment target (Freeman et al., 2020). This is despite evidence that sleep difficulties precede, increase the risk of and exacerbate mental health problems (Hertenstein et al., 2019; McMakin & Alfano, 2015; Orchard et al., 2020); heighten the risk of self‐harm and suicidality (Liu et al., 2019, 2020; Wang et al., 2019) and that behavioural sleep interventions improve mental health across multiple diagnostic presentations (Gee et al., 2019; Harvey et al., 2021; Hertenstein et al., 2022; Scott et al., 2021; Waite et al., 2023).

The unmet sleep need in youth mental health is an area of particular concern, with an especially high prevalence of poor sleep across teenagers and young adults with mental health difficulties (Hysing et al., 2022; Newlove‐Delgado et al., 2022) neurodevelopmental difficulties (Al Lihabi, 2023) and those experiencing childhood adversity (Rojo‐Wissar et al., 2021; Wang et al., 2016).

There is a lack of controlled trials of CBTi in this specialist population, but evidence to date (Åslund et al., 2020; Cliffe et al., 2020; Mathews et al., 2023; Rollinson et al., 2021, 2024; Zetterqvist et al., 2021) consistently suggests a very high level of clinical need, high intervention uptake and completion rates, and good clinical outcomes. Considering this, we have drawn upon implementation science frameworks (Aarons et al., 2011) and recommendations from recent implementation science reviews (Levenson & Williamson, 2023; McGinty et al., 2024; Peters‐Corbett et al., 2024) to guide a programme of work designed to improve access to help with sleep for young people with mental health difficulties.

More specifically, the Exploration, Preparation, Implementation and Sustainment (EPIS) framework (Aarons et al., 2011) outlines four key phases of implementation and identifies external system and internal organizational factors to consider in each phase. Recent reviews of implementation research in mental health (McGinty et al., 2024) and paediatric behavioural sleep interventions (Levenson & Williamson, 2023) advocate for the development of interventions within the complex systems in which they are to be delivered and the use of specific implementation strategies and outcome monitoring (Proctor et al., 2011), as a means of maximizing implementation, sustainability and equality of access. This approach is further reinforced by a systematic review of the implementation of evidence‐based interventions within youth mental health services specifically (Peters‐Corbett et al., 2024). The authors highlight factors such as centralized funding, adapting the intervention to the local context, taking a system‐wide approach to training and the provision of ongoing support, supervision and empowerment as factors found to facilitate successful implementation.

This paper describes a programme of work guided by the EPIS framework that seeks to apply the implementation principles and measures outlined in these reviews to embed improved access to help for poor sleep across a youth mental health system in one rural UK county. Implementation outcomes are first considered for the programme overall and then the CBTi intervention offered within it, followed by an evaluation of the clinical outcomes for the young people receiving the CBTi intervention.

## METHOD

### Implementation strategy

We developed an implementation strategy guided by the following principles: taking a system‐wide approach, embedding evidence‐based interventions within existing routine service delivery, adapting the offer to local service contexts, balancing reach with fidelity, minimizing bureaucratic burden while maintaining effective evaluation and governance, addressing health inequality and planning for sustainability (McGinty et al., 2024; Peters‐Corbett et al., 2024). Table 1 sets out how this was operationalized in practice in relation to the EPIS framework. Data collected up until 21 August 2024 was included in this interim analysis.

**TABLE 1:** Operationalization of EPIS implementation programme phases (Aarons et al., 2011).

**Exploration phase (October 2022 to January 2023)**
Scoping project liaising with over 30 service leads across the integrated care system to establish level of clinical need, perceived need for change and service capacity to accommodate change Explore needs and usual access routes of more vulnerable groups less likely to access a formal therapeutic intervention Compile a business case for central funding from local mental health commissioners
**Preparation phase (January 2023 to September 2023)**
Funding received on annual basis through wider system underspend Establish system‐wide monthly steering group with commissioners able to facilitate co‐ordination and access across service areas Establish accountable and accessible project leadership role Develop bridging roles across clinical and academic departments Contracting with individual services: Identify which practitioners to access which element of the programme; confirm governance and accreditation requirements for therapeutic practitioners (Appendix S1); identify service‐specific sleep leads; confirm outcome measures appropriate to each service and agree information governance arrangements for collection of outcome and implementation data across organizational boundaries Establish online shared access portal for therapeutic practitioners to access therapeutic resources (Future NHS) Run pilot workshops and seek detailed feedback Establish online, inter‐organizational booking system for workshop attendance Communications strategy across service leads and organizational networks to raise awareness of programme and available training
**Implementation phase (February 2023 ongoing (interim analysis August 2024))**
Book practitioners onto online training through liaison with service leads Establish online supervision groups and access to case consultation Monthly monitoring of intervention uptake, measure completion, treatment fidelity, adverse events and training event feedback Incorporate innovation factors/adaptations into the programme and intervention through regular liaison with sleep leads and feedback from supervision groups Develop an inter‐organizational network of sleep leads, with monthly network meeting and sleep seminar Establish central webpage with publicly accessible information, resources and signposting links Programme of awareness raising and attitude change through invited talks
**Sustainment phase (October 2024 ongoing)**
Develop business case for maintenance model to allow ongoing access to training, supervision and workshops Ongoing support for service‐specific sleep leads Move to internal data collection and reporting for all services

### Programme overview

An initial scoping project saw services consistently reporting a very high level of sleep need, poor access to behavioural interventions beyond sleep hygiene, a high perceived need for change and a strong interest in accessing additional training. A programme was subsequently developed comprising two main elements: (i) training for therapeutic practitioners in CBTi adapted for young people with mental health difficulties (The Better Sleep Programme; Rollinson et al., 2021, 2024) and (ii) enhanced skills workshops for non‐therapeutic practitioners, alongside awareness‐raising work and resource development. We also liaised with more specialist services and plan to report on this separately.

#### CBTi training

The CBTi training was presented as an accreditation programme. To achieve accreditation, practitioners were required to attend 2 days of online training (Appendix S2 for overview), 6 months of monthly online supervision, see at least two young people under supervision and collect outcome data. The programme could only be accessed through service managers who were required to nominate a member of staff as a Sleep Lead. A network of Sleep Leads was developed through the provision of monthly seminars and liaison meetings with the programme team.

#### Enhanced skills workshops

Half‐day, online workshops were open to any practitioners working with young people with mental health difficulties. Services working with more vulnerable and socially excluded young people were prioritized to help improve reach and equality of access. Workshop bookings were accepted from individual practitioners or whole services. Workshops sought to raise awareness of the role of sleep in youth mental health, improve skills in identifying sleep problems and increase confidence in offering support, advice and signposting (Appendix S3 for overview). A self‐help guide was developed and shared as a resource attendees could use in practice.

### CBTi intervention

The accreditation programme trained therapeutic practitioners in a formulation‐based CBTi intervention (The Better Sleep Programme), adapted to be appropriate for young people with mental health difficulties and to be delivered by non‐expert practitioners (Rollinson et al., 2021, 2024). Accompanying resources for trained practitioners included a semi‐structured interview outline, a formulation template and resource packs covering the intervention elements outlined in Appendix S4. Emphasis is placed on psychoeducation, stimulus control, boosting sleep drive and using light to regulate circadian rhythm. A low‐intensity form of sleep restriction is used, focusing on going to bed when sleepy‐tired and gradually bringing wake‐up time forward where appropriate. Other sleep disorders are screened for as part of the assessment interview, with signposting advice provided for further assessment where indicated. Six sessions were recommended, with awareness that this may need to be altered for different service settings. A dose of treatment was defined as attending at least two sessions. An intervention was considered completed when outcome measures were returned for the final planned session. Treatment ‘drop out’ was defined as a young person ending *earlier than planned* with their practitioner.

### Participants

Implementation outcomes draw on feedback from practitioners working within the locally commissioned mental health system who attended either the accreditation training or enhanced skills workshops. Practitioners attending the accreditation training were low‐intensity therapeutic practitioners, such as child wellbeing practitioners, psychological wellbeing practitioners and assistant psychologists working across services as outlined in Table 2. Clinical outcomes for the CBTi intervention are reported for 83 service users aged 14–25 years who were accessing a mental health service within the local integrated care system and completed the Better Sleep Programme CBTi intervention with a trained practitioner.

**TABLE 2:** Training and workshop attendance by areas of the mental health system.

Mental health service area	Number attended CBTi training	Routinely offering CBTi	Number attended workshop
Secondary care CFYP core services	33	✓	30
NHS Talking Therapies	70	✓	–
Mental Health Support Teams in schools	34	✓	16
Mental Health Practitioners in PCNs	19	–	7
Voluntary, Community or Social Enterprise	26	✓	116
Local Authority Children's Services	11	✓	75
Secondary care CFYP specialist services	16	N/A	21
Unknown/other	1	N/A	5
Total	210		270

Abbreviations: CFYP: Children, Families and Young People's service; N/A: not appropriate as not included in this analysis; PCNs: primary care networks.

There were no inclusion or exclusion criteria around particular mental health or neurodevelopmental presentations, and information on this could not be reliably collected across services. Appropriateness for a low‐intensity intervention within each service setting was the primary driver for suitability, alongside an ongoing difficulty with sleep and a motivation to work on this.

### Procedure

As outlined in Table 1, therapeutic practitioners were invited to attend the accreditation training through liaison with their service lead after clinical governance, data collection and information governance procedures were agreed. Routinely used wellbeing measures were used where already in place to minimize duplication and bureaucratic burden. Practitioners could submit measures to the programme team by submitting online forms, emailing scanned paper responses or making a record within their own electronic patient record, with anonymized data then shared with the programme team. Training and supervision were hosted online, allowing accurate records of attendance to be collated.

Service users were identified through existing referral processes within each service, although sleep need was often identified during assessment for a different presenting issue. Practitioners were asked to share an information sheet explaining the implementation project and provide confirmation of their client's consent to share data with the programme team. The practitioner delivering the CBTi intervention collected clinical outcome measures at baseline and endpoint. Service users were invited to submit feedback anonymously through an online form during, or shortly after, the final session.

### Design and analysis

A service evaluation approach was taken to reporting implementation and clinical outcomes. The implementation programme is first evaluated against indices of acceptability, adoption and appropriateness, and then the CBTi intervention is evaluated against indices of acceptability, accessibility, fidelity and appropriateness. Clinical outcomes for the CBTi intervention are examined using a within‐subject design to compare measures of sleep, wellbeing and progress toward personal goals taken at assessment (baseline) and final session (endpoint).

### Ethical considerations

Information sharing arrangements were agreed with each service accessing the accreditation programme during the preparation phase of the implementation strategy (Table 1). Data was submitted anonymously and with service user consent (parental consent or an assessment of Gillick competence (Department of Health, 2009) was required for those under 16 years). Withholding consent did not affect access to the intervention. The service evaluation was registered with and approved by the Norfolk and Suffolk NHS Foundation Trust Research and Development department (2023MH10‐SE).

### Measures

#### Implementation outcomes

##### Programme evaluation

Acceptability of the programme was evaluated through training and workshop attendance, feedback, supervision attendance and sleep lead engagement.

Adoption across the local system was evaluated in terms of the proportion of trained therapeutic practitioners able to deliver the intervention locally, the number of participating services offering the CBTi intervention as part of their routine offer and the estimated number of young people and families receiving additional support with their sleep from non‐therapeutic practitioners.

Appropriateness of the programme was assessed in reference to any adaptations to the programme structure needed over time or across different service contexts.

##### CBTi intervention evaluation

Acceptability of the CBTi intervention was evaluated through intervention and measure completion rates and responses on programme‐specific service user and practitioner feedback forms. Service users were asked to provide feedback on the intervention generally and to rate the amount of support they felt they needed and the ease with which they were able to access it. Practitioners were asked to report on the number of sessions delivered, session content, medium used, adverse events, the perceived helpfulness of the intervention, the level of support required following the intervention, whether the young person ended sessions earlier than planned and possible reasons why.

Accessibility was evaluated by considering the demographic characteristics of service users accessing and completing the intervention. Fidelity was assessed through practitioners' end of treatment ratings of session content using an adherence checklist. Appropriateness was assessed by reference to any adaptations needed across different service contexts.

#### Clinical outcomes

Clinical outcome measures were completed by the young person except for the summary table and calculation of the Sleep Efficiency Quotient, which were completed by the practitioner based on the young person's verbal report and sleep diary data, respectively.

##### Sleep measures

###### Insomnia severity index (ISI; Bastien et al., 2001)

The ISI is a seven‐item self‐report questionnaire used to detect insomnia in community, clinical and adolescent populations (Chung et al., 2011; Gagnon et al., 2013; Gerber et al., 2016; Morin et al., 2011). Higher scores represent greater symptoms of insomnia.

###### Sleep efficiency quotient (SEQ)

The SEQ (Reed & Sacco, 2016) reflects the proportion of time spent in bed that is spent sleeping and is expressed as a percentage. It is calculated from sleep diaries (adapted from the consensus sleep diary (Carney et al., 2012) to be appropriate for a youth population). Where a sleep diary was not available, a retrospective account of the previous three nights was accepted.

###### Sleep summary table (Appendix S5)

A sleep summary table was developed for completion at the end of the assessment interview and final session. The table allows a standardized description of the presenting sleep difficulty and screens for other sleep disorders. The practitioner completed this table based on self‐reported information gathered from the young person. Total sleep time and sleep onset latency were analysed.

##### Wellbeing measures

Wellbeing measures being routinely collected by services were used where available, resulting in multiple indices of wellbeing.

###### Clinical Outcomes in Routine Evaluation‐10 (CORE‐10; Barkham et al., 2013)

The CORE‐10 is a 10‐item self‐report measure designed to assess psychological distress. The scale is derived from the CORE‐OM (Evans et al., 2002) and comprises items on anxiety, depression, trauma, physical problems, functioning and risk. Higher scores indicate greater levels of psychological distress.

###### Young Person's Clinical Outcome in Routine Evaluation (YP‐CORE; Twigg et al., 2009)

The YP‐CORE is a 10‐item self‐report version of the CORE‐10 developed to evaluate psychological distress in adolescents (11–16‐year‐olds) (Twigg et al., 2009).

###### The Revised Child Anxiety and Depression Scale (RCADS: Chorpita et al., 2000)

The RCADS is a 47‐item self‐report measure designed to assess symptoms of anxiety and low mood in young people (Chorpita et al., 2000, 2005; Piqueras et al., 2017). Higher scores indicate greater symptoms of anxiety and low mood.

###### Patient Health Questionnaire‐9 (PHQ‐9; Kroenke et al., 2001)

The PHQ‐9 is a nine‐item self‐report scale designed to screen and monitor the severity of depression. The measure is routinely used in clinical practice and is well validated (Kroenke et al., 2010; Löwe et al., 2004; Wittkampf et al., 2007). Higher scores indicate greater levels of depression.

###### General Anxiety Disorder‐7 (GAD‐7; Spitzer et al., 2006)

The GAD‐7 is a seven‐item self‐report scale designed to assess anxiety. It is routinely used in clinical practice and has good psychometric properties (Löwe et al., 2008; Spitzer et al., 2006). Higher scores indicate greater levels of anxiety.

##### Progress toward personal goals

The goal based outcomes (GBO; Law & Jacob, 2013) are a well‐established tool used to set and track progress toward up to three goals (Edbrooke‐Childs et al., 2015; Jacob et al., 2017). Service users identify goals at assessment and re‐evaluate them during their final session, with progress toward each goal rated out of 10. An average score is computed.

### Analysis plan

The implementation measures for the wider programme and the CBTi intervention were reported using descriptive statistics.

Clinical outcomes were then evaluated by examining the change from baseline to endpoint using one‐tailed paired *t*‐tests and the proportion of service users meeting criteria for clinical thresholds.

## RESULTS

### Implementation outcomes

#### Programme evaluation

##### Acceptability

A total of 210 practitioners from 18 services had attended the accreditation training at the time of analysis, reflecting 88% of all invitations. Feedback was received from 83% (*n* = 175) of training attendees. Of these, 81% (*n* = 141) rated the training as ‘very interesting,’ 98% (*n* = 171) reported gaining new knowledge about sleep and said they would recommend the training to a colleague.

In terms of acceptability of the workshops, 270 staff from 29 services had attended at the time of analysis, reflecting 67% of bookings made, with 95% (*n* = 213/224) of attendees that provided feedback saying they would recommend the workshop to a colleague. At the time of analysis, 9 of the 11 workshops scheduled over the next 6 months were fully booked.

A total of 20 CBTi supervision groups were established, with 10 ongoing. 90% (*n* = 109/121) of active clinical practitioners offered supervision attended at least one session. The overall attendance rate for the 10 completed groups was 62%.

Twenty‐one sleep leads were identified, reflecting at least one per service that attended the training. The average attendance of sleep leads at the monthly seminars was 58%.

##### Adoption

**FIGURE 1:**
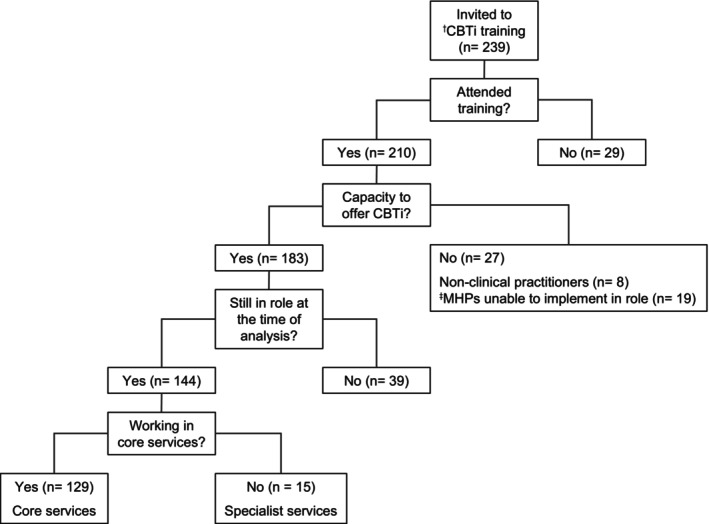
Flow diagram of trained practitioners. ^†^CBTi, Cognitive Behavioural Therapy for Insomnia; ^‡^MHPs, Mental Health Practitioners in Primary Care Networks (PCNs).

Prior to the implementation programme, there were three practitioners in the Secondary Care core service still in post who had been trained as part of a previous service evaluation (Rollinson et al., 2024). There were otherwise no trained practitioners routinely offering CBTi within the local services listed. Figure 1 outlines the number of trained therapeutic practitioners able to offer the CBTi intervention following the implementation programme. Table 2 indicates the number of therapeutic practitioners in each area of the mental health system who had attended the accreditation training or workshops and which service areas were offering CBTi as part of their routine service offer at the time of writing. All services approached to engage with the accreditation programme did so.

At least 136 14–25‐year‐olds were offered the sleep intervention as a standalone, formal intervention. Informal feedback in supervision suggests that many more young people were offered advice about sleep as part of another primary intervention without formal sleep measures being collected.

Of the 270 workshop attendees, 86% (*n* = 193/225) rated themselves as ‘highly likely’ to apply their new knowledge on sleep in practice. The 181 respondents with codable data estimated planning to work with an average of 7 young people from their *current* caseload on sleep as a result of the workshop.

##### Appropriateness

The two main elements of the implementation programme, the CBTi training and the workshops, were appropriate for most services approached. One exception was the Mental Health Practitioner service that offers low‐intensity interventions in a primary care setting. Changes to the service context limited its ability to offer consistent sessions of sufficient frequency, so the self‐help guide was agreed to be a more appropriate means of sleep support.

Other adaptations and innovations included the use of a wide range of wellbeing measures to maximize adoption within services, additional support for staff more recently qualified and a shorter training for staff in a purely supervisory or strategic role. Changes were also made to existing referral processes across multiple services to allow poor sleep to be identified at assessment and support to be accessed appropriately.

#### CBTi intervention evaluation

##### Acceptability

Of the 165 young people who had started the intervention at the time of analysis, 136 were within the target age range and of these, 16 were still in treatment. Of the 120 no longer in treatment, 83 (69%) had completed the intervention and returned paired outcome data. Two young people had completed the intervention, but their practitioner had left the service before submitting their endpoint data.

A total of 35 young people (29%) had ended the intervention earlier than planned (treatment dropout) and not returned endpoint measures. When asked about the likely reasons for ending early, practitioners most frequently cited wider life events (*n* = 10) or a loss of contact (*n* = 9). Other reasons involved an improvement in their sleep (*n* = 3), worsening in mental health difficulties (*n* = 5), other needs being prioritized (*n* = 4) or not liking the sleep intervention (*n* = 2).

Practitioners reported 23 adverse events; however, upon review, only 13 were categorized as adverse. Of these, one was considered related to the intervention (sleep medication was stopped toward the end of the intervention, resulting in some side effects). Examples of events listed by practitioners as adverse but considered not to reach the threshold included the school holidays causing a pause in sessions, a service user's mother splitting up from her partner and a change of practitioner due to the previous one leaving.

End of treatment reports were received for 111 of the 136 clients who started the intervention. These indicated that the intervention was delivered over an average of 4.75 sessions (SD = 2.49 [mean of 2.03 in those that dropped out and 5.90 for those that completed the intervention]). The majority (*n* = 71, 64%) of young people met with their therapist face‐to‐face, with 29 (26%) meeting remotely (telephone or video) and 11 (10%) using a combination of these.

The average outcome measure completion rate for all service users that submitted baseline data (*n* = 136) was 84% (range 57% to 96%) at baseline and 56% (range 41% to 64%) at endpoint. The measure completion rate at endpoint for those that returned endpoint data (*n* = 83) was 79% (range 58% to 96%).

Anonymized feedback was received from 36 service users. Overall, 83% (*n* = 30) would recommend the intervention, with 36% (n = 13) feeling they needed less support and 69% (*n* = 24/35) finding it easier to make use of the support available to them. Practitioner feedback corroborated this, with 73% (*n* = 58/80) considering the intervention to have been helpful to the service user they were working with and 42% (*n* = 47/111 responses) reporting the service user needed less or no further support following the intervention.

##### Accessibility

Table 3 shows the demographic characteristics of the 136 14‐ to 25‐year‐olds who accessed the CBTi intervention. The majority (72%) identify as female and White (92%) and in some form of regular structured employment or activity (74%). The lack of ethnic diversity is reflective of the local population (UK Census, 2021).

**TABLE 3:** Demographic characteristics of those accessing the CBTi intervention.

	*n*	*%*
**Biological sex**		
Male	23	18
Female	101	80
Prefer not to state	3	2
*n* = 127		
**Identifies as**		
Male	25	20
Female	91	72
Non‐binary	6	5
Agender/genderless	1	1
Prefer not to state	3	2
Did not identify with the above	1	1
*n* = 127		
**Ethnicity**		
White–British	109	86
White–any other white background	7	6
Mixed‐white and Asian	1	1
Asian/Asian British	3	2
Black/Black British	1	1
Prefer not to state	1	1
Not known	5	4
*n* = 127		
**Education/employment**		
In full time education/employment	66	56
In part time education/employment	19	16
Other regular structured activity	2	2
Not in any regular structured activity	25	21
Not known	6	5
*n* = 118		

A comparison was made of demographic characteristics and baseline sleep and personal goal measures between young people who completed the intervention (*n* = 85; 2 of whom did not provide endpoint data) and those who did not (*n* = 35). The groups significantly differed in practitioner‐estimated sleep onset latency (SOL), with those who completed the intervention reporting lower SOL (2.57 h, SD = 1.84, *n* = 70) than those who did not complete (3.53 h, SD = 2.0; *n* = 24, *p* = 0.01). No differences in gender, ethnicity, age, ISI score or other baseline characteristics were identified between completers and non‐completers (Appendix S6 for further details).

##### Fidelity

End of treatment practitioner reports were received for 78 of the 83 clients with paired clinical outcomes. No interventions were described as using sleep hygiene only (psychoeducation and quick wins), with 97% (*n* = 76) using at least one sleep‐specific strategy. Most interventions (77%, *n* = 60) used a combination of sleep hygiene, sleep‐specific strategies, and cognitive and behavioural strategies.

##### Appropriateness

Adaptations were made to the CBTi intervention where needed to maximize adoption in practice without interfering with the core content. A large resource pack was quickly replaced by booklets covering specific elements of the intervention, and a formulation worksheet with assessment prompts helped more experienced staff identify maintaining factors more efficiently. One service needed more text‐based resources and a more structured approach to session planning due to shorter session times. Another service was limited to offering young people a single intervention, so a pathway was developed that allowed three sessions of CBTi to sit alongside a mental health intervention.

### Clinical outcomes

Clinical outcomes are reported for the 83 service users aged 14‐to‐25‐years with paired data.

Table 4 summarizes the descriptive and inferential statistics for the CBTi intervention clinical outcome measures. Paired *t*‐tests revealed statistically significant differences between baseline and endpoint scores across all outcome measures with moderate to large effect sizes (Cohen, 1988).

**TABLE 4:** Descriptive and inferential statistics for CBTi intervention outcome measures (*n* = 83).

Measure	*n*	Baseline	Endpoint	Mean difference	*d* (CI)
		Mean (SD)	Mean (SD)		
**Sleep measures**					
ISI	75	17.90 (4.75)	10.41 (5.62)	7.48***	1.35 (1.01, 1.68)
SEQ	47	63.20 (15.85)	80.32 (13.16)	−17.13***	−1.03 (−0.63, −1.43)
SOL	33	2.89 (1.82)	1.52 (1.27)	1.37***	1.17 (0.87, 1.47)
TST	47	5.97 (2.04)	7.06 (1.48)	−1.09***	−0.61 (−0.31, −0.92)
**Wellbeing measures**					
YP‐CORE	25	20.44 (5.80)	14.28 (6.13)	6.16***	1.26 (0.83, 1.69)
CORE‐10	26	23.89 (8.88)	17.15 (9.08)	6.74***	0.92 (0.55, 1.30)
RCADS	10	65.30 (15.90)	57.70 (16.90)	7.60*	0.88 (0.49, 1.26)
PHQ‐9	10	12.60 (6.69)	7.70 (3.30)	4.90**	0.91 (0.26, 1.57)
GAD‐7	11	9.00 (6.25)	6.18 (4.85)	2.82*	0.57 (0.02, 1.12)
**Progress toward goals**					
GBO	71	1.78 (1.49)	6.20 (2.34)	−4.42***	−1.77 (−1.30, −2.24)

Abbreviations: CI, lower and upper 95% confidence interval; CORE‐10, clinical outcomes in routine evaluation‐10; *d*, Cohen's *d*; GAD‐7, general anxiety disorder‐7; GBO, goal based outcomes; ISI, insomnia severity index; PHQ‐9, patient health questionnaire‐9; RCADS, revised children's anxiety and depression scale; SEQ, sleep efficiency quotient; SOL, sleep onset latency in hours; TST, total time asleep a night in hours; YP‐CORE, young person's clinical outcome in routine evaluation.

*p < .05. **p < .01. ***p < .001.

Table 5 summarizes the change in percentage of service users meeting clinical thresholds at baseline and endpoint for the sleep and wellbeing measures.

**TABLE 5:** Clinical threshold analysis.

Measure	Clinical threshold	% at baseline	*n*	% at endpoint	*n*
ISI	≥15	77	58	21	16
YP‐CORE	≥14.1 for males, ≥15.9 for females	88	22	32	8
CORE‐10	≥11	85	22	69	18
RCADS	≥70	60	6	40	4
PHQ‐9	≥10	60	6	20	2
GAD‐7	≥8	55	6	18	2

*Note*: Clinical thresholds: ISI Bastien et al. (2001); YP‐CORE Twigg et al. (2016); CORE‐10 Barkham et al. (2013); RCADS Chorpita et al. (2000); PHQ‐9 Kroenke et al. (2001); Kroenke et al. (2010); Manea et al. (2012); Manea et al. (2015); GAD‐7 Kroenke et al. (2007); Plummer et al. (2016); Johnson et al. (2019).

When combining all wellbeing measures, 76% of service users were above the clinical threshold at baseline, with this dropping to 41% at endpoint.

Reliable change was observed in 82% (*n* = 58) of service users completing the GBO (≥2.45‐point difference; Edbrooke‐Childs et al., 2015).

## DISCUSSION

### Summary of findings

We saw very high levels of service engagement with both the training and workshop elements of the implementation programme, alongside positive clinical outcomes in both sleep and wellbeing in the young people receiving the CBTi intervention.

The CBTi accreditation training was accepted by all services approached. The high levels of service engagement were reflected in high training and workshop uptake, good attendance at supervision, high rates of accreditation and ongoing engagement with a network of sleep leads.

The positive clinical outcomes from the CBTi intervention previously seen in secondary care (Rollinson et al., 2024) were maintained at this wider scale with statistically significant improvements and large effect sizes reported in all measures of sleep and well‐being. The proportion of young people meeting the threshold for clinical insomnia fell from 77% at baseline to 21% at endpoint, with 69% of the 35 providing feedback reporting feeling better able to make use of available support.

These findings are in‐keeping with randomized controlled trials (RCTs) and systematic reviews reporting improvements in sleep to be associated with more modest but significant improvements in mental health measures (Freeman et al., 2017; Friedrich et al., 2018; Gee et al., 2019; Harvey et al., 2021; Hertenstein et al., 2022; Scott et al., 2021; Waite et al., 2023). It is encouraging to see these improvements replicated within a routine youth mental health care setting.

### Clinical implications

The implementation science approach outlined in Table 1 (Levenson & Williamson, 2023; McGinty et al., 2024; Peters‐Corbett et al., 2024) has been successful to date in improving access to help with sleep across a wider mental health system. With 129 therapeutic practitioners available to offer the CBTi intervention, it is now routinely available in five of the six core mental health services we worked with. The clinical improvements reported to date are particularly striking given the variety of low‐intensity practitioners working across different service areas and the transdiagnostic offer across many mental health presentations.

Three factors that have seemed particularly key to the programme's success have been its centralized funding, the provision of support and supervision after the CBTi training and the willingness to adapt the CBTi intervention to fit local service parameters.

The current programme has been running for 18 months at the time of writing, and the sustainability of any change is yet to be established. Factors to date that have seemed key to promoting sustainability have been the development of a network of local sleep expertise in the 21 service‐specific sleep leads, embedding the offer of a sleep intervention in existing referral and governance processes and raising awareness more broadly to allow sleep need to be identified and assessed across the mental health system.

An alternative implementation strategy may be to build on the positive clinical outcomes seen using a digital CBTi intervention within adolescent mental health services (Cliffe et al., 2020; Mathews et al., 2023; Zetterqvist et al., 2021) and the clear advantages this offers in terms of accessibility and efficiency. It will be interesting and important to compare an implementation approach focused on making a CBTi intervention available (be it digital or in‐person) with one that also incorporates a programme of awareness raising. We have taken the stance that improving access to help with poor sleep goes beyond accessing a therapeutic intervention, and the half‐day workshops have seemed an important element of the strategy in this regard. Further work is needed, however, to better understand the impact of these sessions on clinical practice.

### Limitations

This is a real‐world service evaluation of an implementation strategy rather than a controlled clinical trial of an intervention. There are multiple sources of potential bias with therapists collecting outcome data, training providers collecting feedback, no control comparison, a self‐selecting sample of service users providing feedback and only subjective measures of sleep available. We were also unable to control for the influence of other interventions that may have been accessed. Multiple wellbeing measures were used to maximize implementation in routine practice, limiting the sample size of some measures where services were relatively recently trained. While most service users who started the intervention did complete it, almost a third did not, and we are not able to account for their outcomes. The positive clinical outcomes reported are therefore likely inflated, and the lack of follow‐up data means we cannot know the extent to which improvements were maintained. It is also difficult to extrapolate the impact of an accreditation programme requiring practitioners to see cases on the uptake of the intervention by services. Longer‐term monitoring of the sustainability of the programme will be critical, as will establishing its generalizability.

### Future directions

Youth mental health services in the United Kingdom are well placed to deliver a low‐intensity CBTi intervention given the large workforce of low‐intensity practitioners (Ludlow et al., 2020) and the relative homogeneity of sleep difficulties in a younger clinical population (Baddam et al., 2018; Reeve et al., 2019). To fully embed help with sleep as an integral part of youth mental health services, however, implementation programmes such as these need a commitment to ongoing funding to allow the current offer to be maintained, CBTi training needs to be included within the curricula for low‐intensity practitioners and sleep awareness needs to be incorporated as an induction/training requirement for staff across the mental health system.

Further studies examining any variation in implementation and clinical outcomes across mental health services would help target future implementation work more efficiently, as would a more detailed understanding of the reach of any awareness‐raising work into more traditionally excluded communities and clinical groups. A stronger service user voice would strengthen any future developments, as would a more detailed understanding of the type of sleep difficulty experienced by young people accessing mental health services.

More research is clearly needed in this area to consider clinical and health economic outcomes when addressing comorbid sleep and mental health conditions. The finding that service users felt better able to engage with available support following a sleep intervention, for instance, suggests huge potential for improved sleep to help mediate improved engagement with mental health‐specific interventions and warrants careful health economic evaluation.

## CONCLUSIONS

Young people with mental health difficulties are reporting high levels of insomnia and a willingness to work on their sleep, but evidence‐based interventions are rarely offered routinely. Interim outcomes of an implementation strategy that emphasizes taking a whole‐system approach and adapting interventions to fit specific populations and service contexts indicate that services are keen to provide a sleep intervention and are seeing positive clinical outcomes when supported to do so. Further evaluation is needed to establish the sustainability and generalizability of this work.

## AUTHOR CONTRIBUTIONS

**Rebecca Rollinson:** Conceptualization; investigation; funding acquisition; writing – original draft; methodology; writing – review and editing; visualization; formal analysis; resources; data curation; project administration. **Ben Ewing:** Investigation; writing – original draft; formal analysis; project administration; data curation; resources; writing – review and editing; validation. **Sarah Reeve:** Writing – review and editing; formal analysis; methodology; investigation; supervision. **Adam Graham:** Investigation; conceptualization; writing – review and editing; methodology; project administration; resources. **Jonathan Lyons:** Conceptualization; investigation; writing – review and editing; visualization; supervision; project administration; resources. **Brioney Gee:** Conceptualization; writing – review and editing; methodology; visualization; supervision. **Jonathon Wilson:** Conceptualization; writing – review and editing; visualization; supervision. **Ioana Tofan:** Writing – review and editing; project administration; investigation; methodology. **Kelly Semper:** Conceptualization; funding acquisition; writing – review and editing; methodology; supervision. **Tim Clarke:** Conceptualization; methodology; writing – review and editing; supervision.

## FUNDING INFORMATION

This project was funded by the Norfolk and Waveney Integrated Care Board.

## CONFLICT OF INTEREST STATEMENT

The authors declare that they have no conflicts of interest.

## Supporting information


Data S1.

## Data Availability

Data sharing is limited by information governance agreements. Contact the corresponding author for further information.
