# Supplementary material for: Improving access to help with poor sleep across youth mental health services: Interim implementation and clinical outcomes
Source: Br J Clin Psychol. 2025 Mar 14;65(3):844–61. doi: 10.1111/bjc.12531 (PMC13456474; doi:10.1111/bjc.12531)
Supplement: Supplementary file 1 [file BJC-65-844-s001.docx]

**Appendix S1**

Governance guidelines for accreditation programme

**Appendix S2**

Overview of CBTi training days

**Appendix S3**

Overview of the Bite-Sized Better Sleep Workshops

**Appendix S4**

Overview of the CBTi Intervention

**Appendix S5**

Sleep summary table

**Appendix S6**

Comparison of age and baseline measures in those that completed the intervention and those that dropped out.

| Baseline characteristic | Completed CBTi (n=85) | | Dropped out of CBTi (n=35) | | Test statistic | p |
| --- | --- | --- | --- | --- | --- | --- |
|  | Descriptor | n | Descriptor | n |  |  |
| Female biological sex (%) | 77.11 | 83 | 81.25 | 32 | 0.02^a^ | 0.88 |
| Identifies differently to biological sex (%) | 10.84 | 83 | 9.38 | 32 | 0.05 ^a^ | 0.82 |
| Face to face sessions (%) | 75.64 | 78 | 66.67 | 33 | 0.43 ^a^ | 0.52 |
| Ethnicity: Non-white (%) | 6.02 | 83 | 0 | 34 | 2.54 ^a^ | 0.28 |
| Age (sd) | 18 (3.32) | 85 | 18.11 (3.42) | 35 | 1479^b^ | 0.96 |
| ISI (sd) | 17.82 (4.64) | 83 | 18.76 (3.82) | 33 | 1192 ^b^ | 0.30 |
| SEQ (sd) | 62.08 (18.91) | 58 | 53.68 (23.72) | 10 | 220 ^b^ | 0.23 |
| GBOM (sd) | 1.76 (1.47) | 75 | 1.88 (1.71) | 28 | 1022 ^b^ | 0.83 |
| SOL (sd) | 2.57 (1.84) | 70 | 3.53 (2.0) | 24 | 540 ^b^ | 0.01 |
| TST (sd) | 5.80 (1.97) | 72 | 5.77 (1.98) | 31 | 1069 ^b^ | 0.74 |

Note: ISI = Insomnia Severity Index. SEQ = Sleep Efficiency Quotient. GBO = Goal Based Outcomes. SOL = Sleep onset latency in hours. TST = Total time asleep a night in hours.

^a^ Chi Square, ^b^ Mann-Whitney U was used as the assumption of normality was not met in 3 variables.
